# Informed consent practices for surgical care at university teaching hospitals: a case in a low resource setting

**DOI:** 10.1186/1472-6939-15-40

**Published:** 2014-05-19

**Authors:** Joseph Ochieng, Charles Ibingira, William Buwembo, Ian Munabi, Haruna Kiryowa, David Kitara, Paul Bukuluki, Gabriel Nzarubara, Erisa Mwaka

**Affiliations:** 1 Anatomy Department, School of Biomedical Sciences Makerere University, P.O 7072, Kampala, Uganda; 2 Surgery Department, Gulu University, Gulu, Uganda; 3 Department of Social Administration College of Humanities and Social Sciences, Makerere University, Kampala, Uganda; 4 Anatomy Department, Kampala International University, Ishaka, Uganda; 5 Anatomy Department, St. Augustine International University, Kampala, Uganda

**Keywords:** Informed consent practices, Surgery, Uganda

## Abstract

**Background:**

Informed consent in medical practice is essential and a global standard that should be sought at all the times doctors interact with patients. Its intensity would vary depending on the invasiveness and risks associated with the anticipated treatment. To our knowledge there has not been any systematic review of consent practices to document best practices and identify areas that need improvement in our setting. The objective of the study was to evaluate the informed consent practices of surgeons at University teaching Hospitals in a low resource setting.

**Methods:**

A cross-sectional study conducted at three university teaching hospitals in Uganda. Self-guided questionnaires were left at a central location in each of the surgical departments after verbally communicating to the surgeons of the intention of the study. Filled questionnaires were returned at the same location by the respondents for collection by the research team. In addition, 20 in-depth interviews were held with surgeons and a review of 384 patients’ record files for informed consent documentation was done.

**Results:**

A total of 132 (62.1%) out of 214 questionnaires were completed and returned. Respondents were intern doctors, residents and specialists from General surgery, Orthopedic surgery, Ear, Nose and Throat, Ophthalmology, Dentistry, Obstetrics and Gynaecology departments. The average working experience of respondents was 4.8 years (SD 4.454, range 0–39 years). 48.8% of the respondents said they obtained consent all the time surgery is done while 51.2% did not obtain consent all the time. Many of the respondents indicated that informed consent was not obtained by the surgeon who operated the patient but was obtained either at admission or by nurses in the surgical units. The consent forms used in the hospitals were found to be inadequate and many times signed at admission before diagnosing the patient’s disease.

**Conclusions:**

Informed consent administration and documentation for surgical health care is still inadequate at University teaching hospitals in Uganda.

## Background

Informed consent in medical practice is essential and a global standard that should be sought at all the times doctors interact with patients [1-10]. It’s intensity varies depending on the invasiveness and risks associated with the anticipated treatment [11]. The importance attached to ethical practice and associated informed consent varies from doctor to doctor and this is further influenced by working environment, level of knowledge, experience and societal values and beliefs. In spite of these factors, patients’ autonomy should be respected as doing so is actually respecting the patients’ rights as an individual to make decisions that affect their lives [11-15]. It should be noted that respecting individual’s rights is one way to ground respect of autonomy.

Surgery is an invasive practice and would require a more rigorous informed consent process for improved shared decision making because of the higher frequency and intensity of associated risks as compared to general medical care. Since Surgery is performed on a routine basis at the University teaching hospitals, surgeons should be well grounded in ethical and professional practice so as to role model for their students.

While a number of studies on informed consent have been carried out in the region in the recent past, they have focused on informed consent during research [16-21]. To our knowledge there has not been any systematic review of informed consent practices for surgical care, and thus no documented best practices. This study was intended to evaluate what the surgeons at teaching hospitals do regarding informed consent because it impacts their trainees’ knowledge and practice.

## Methods

This was a cross-sectional study conducted at three university teaching hospitals in Uganda. A surgeon was defined as any doctor (specialist or not) who routinely performed surgical procedures. Data was collected using self administered questionnaires, in-depth interviews and, evaluation of the informed consent process and documentation in these hospitals.

The self administered interview used a semi-structured questionnaire that employed a mainly qualitative approach (Additional file 1). Questions in this questionnaire were adopted from previous studies [16,17]. It included questions like what constituted informed consent, how it is obtained, whether obtained at all times, who obtains and what type of information is exchanged between doctors and their patients. In-depth interviews were done purposively to seek clarification on the information that had been captured by the returned questionnaires (Additional file 2). In addition 384 patients files randomly selected, were reviewed for documentation of the informed consent process to evaluate if patients’ condition was clearly confirmed to the patient including diagnosis, proposed procedures, risks and benefit of the treatment as well as long term effects following treatment. It also looked at clear recording of the name of the surgeon in charge and the person who obtained consent. The records review was intended to back up information captured from the questionnaires [16]. Two hundred fourteen questionnaires and informed consent forms were distributed to the surgical units of the respective University teaching Hospitals. Surgeons from the following surgical departments participated in the study; General surgery, Orthopedic surgery, Ear-Nose-Throat, Ophthalmology, Dentistry, Obstetrics and Gynaecology. Participants were given ample time of at least one week to fill the questionnaires and reminders sent to those who delayed to return the filled questionnaires.

### Data management

Qualitative data was collected and recorded in note form. Data was checked in the field to ensure that all the information had been properly collected and recorded. This process was repeated to ensure completeness and consistency. Thematic and content analysis was carried out whereby field notes were categorized according to the research themes and interpreted in line with the study objectives and research questions. Relevant comparisons were made between the different groups of informants.

The survey data was entered into Epidata version 3.2 (Epidata association, Denmark) and exported to SPSS version 17 where after checking for duplicate entries a preliminary analysis of each variable was made to identify additional range and omission errors. Averages for questions that required quantitative answers and frequency tables for questions that required a choice among several given alternatives were calculated.

### Ethical considerations

Ethical approval was sought from the Makerere University School of Biomedical Sciences Research and Ethics Committee and the Uganda National Council for Science and Technology. Permission to conduct the study was obtained from the respective hospital administrations. Informed consent was obtained before recruitment of any participant into the study while participant’s identifying information was kept confidential.

## Results

One hundred thirty-three of two hundred fourteen questionnaires were completed and returned, 384 patients’ record files were reviewed and 20 in-depth interviews conducted.

The response rate for the questionnaires was 62.1% with mean age of the surgeons at 33.1 years (SD 8.420, range 22 to 64 years). There were more males (80.5%) than females (19.5%). About 60.2% had a bachelors’ medical degree, 33.8% had masters’ degrees and 3% had doctorate degrees. The respondents had a mean working experience of 4.8 years (SD 6.454, range 0–39 years). About 29.3% of the respondents were intern doctors or residents in a surgical discipline, 16.5% were from general surgery, 16.5% from Obstetrics and Gynaecology, 9.8% from dentistry, 13.5% from orthopedics, 6% from ophthalmology, while 4.5% were general practitioners.

The average number of patients operated per participant was 10.4 (SD 20.2) per week.

Though 99.2% of respondents agreed that informed consent was necessary before any surgical procedure only 48.8% reported routinely obtaining consent all the time they performed surgery (Table 1). There were varying responses concerning the timing of the informed consent process with majority (42.1%) saying that informed consent should be obtained before any surgical procedure (Table 2).

**Table 1:** Cross tabulation of obtaining informed consent all the time by specialization

**Specialty**	**Yes (%)**	**No (%)**	**Total**	**% of overall total**	**Odds ratio**
Intern doctors	18 (50)	18 (50)	36	28.8	1
Specialists	21 (44.7)	26 (55.3)	47	37.6	0.81 (0.31 to 2.11)
Postgraduates	10 (41.7)	14 (58.3)	24	19.2	0.71 (0.22 to 0.28)
General practioners	12 (66.7)	6 (33.3)	18	14.4	2 (0.54 to 7.92)
**Total**	**61 (48.8)**	**64 (51.2)**	**125**	**100**	

The general practioners included both medical officers and dental surgeons with a bachelors degree and had completed internship.

**Table 2:** When informed consent should be sought

	**Frequency**	**Percent**
Before any procedure	56	42.1
When patient is fully conscious	9	6.8
On admission	5	3.8
Elective and emergency surgery	10	7.5
Soon after examination	14	10.5
For complex treatment	19	14.3
Anytime	7	5.3
Medical research	2	1.5
Above 18 with stable mind	1	0.8
On change of drugs	1	0.8
Several treatment options	1	0.8
24 Hrs before procedure	2	1.5
Not answered	6	1.5
**Total**	**133**	**100**

88.6% of the respondents obtained informed consent from patients at their last surgical operation. 34% of the respondents did not know the definition of informed consent. In response to who to secure consent, majority thought that informed consent should be obtained by the surgeon performing the procedure though a number of others believed that a nurse or any other qualified health worker on the ward where the patient is admitted should obtain informed consent. Other responses included; informed consent was obtained by the admitting nurse/doctor; by signing the consent form or is obtained verbally or implied consent.

All hospitals included in the study did not have proper consent forms. The only evidence of document consent was the admission form signed by all patients at admission for in-patient care. Signing of the admission form in many cases occurs before a patient’s diagnosis is established and thus no treatment plan is included. Informed consent documentation in the patient’s records was most of the times inadequate.

In response to how much information should be availed to the patient during the informed consent process, most of the surgeons responded that patients should be given enough information and nothing should be withheld. Respondents suggested that the following information should be included on the information sheet of the consent form: the patients’ condition, management options, all treatment and surgical procedure risks involved, benefits of the procedure, details of the operation, outcome and possible complications as much as a patient can understand.

## Discussion

The estimated number of doctors working in the surgical units at the three teaching hospitals was 214 which was the number of questionnaires handed out. A total of 132 (62.1%) out of 214 questionnaires were completed and returned which is better than a 52% recorded in a previous related study [19]. Another related study gave a response rate of 63% [22]. This also highlights the fact that staff at university teaching hospitals in Uganda tend to be reluctant to respond to surveys.

The average age of the surgeons was 33.1 years. The average age was low because many of the respondents were either doctors doing their internship or graduate students pursuing their masters degree training. In addition, the majority of the employees at the university and associated teaching hospitals are actually in the age group 30–40 years [19]. Medical doctors in Uganda are usually at least 25 years of age at the time of qualification if trained in Uganda. However, since the surgical units have clinical officers who participate in surgery, the four respondents with a diploma also contributed to the younger age of 22 and these are clinical officers who participate in surgical procedures at the hospitals.

The 60.2% of respondents with Bachelor’s degree was high because many of the respondents were either intern doctors or postgraduate students who tend to respond to surveys better than their senior counter parts. In addition, they all perform surgery and contribute to teaching of medical students in our setting. There were no significant differences in obtaining informed consent among doctors among the different specialties, levels of education or experience as shown in Table 1.

Obtaining informed consent among respondents was seen improving among those from internship to those in general practice but then went down during graduate training. Also specialists were more than 20% less likely to obtain informed consent as compared to intern doctors (Table 1).

There were more males than females which are a reflection of the fact that most of the surgery based clinicians are actually male. There are relatively fewer females in the surgical specialties in Uganda.

The average number of patients operated per week was 10.4 and this is because of the high burden of disease and low numbers of doctors in our setting, hence increased work load for the few available doctors at public hospitals [23-25].

Almost all, (99.2%) of the respondents agreed that informed consent is necessary though only 48.8% reported obtaining consent all the time surgery is done. Despite the fact that emergency surgery occurs at these hospitals, the magnitude of such operations with no next of kin available to give informed consent cannot be as high as 51.2% meaning that sometimes doctors do not obtain informed consent from their patients with no genuine reason (Table 1). It is also true that for emergency surgery with no available next of kin, the senior hospital administration should consent on behalf of the patients. In this study, 88.6% of the respondents said they had obtained informed consent at their last surgical operation. However, the quality of such consent may be lacking since no adequate documentation of informed consent was available from reviewed patient records.

There was a big variation among respondents as to when consent should be sought (Table 2) indicating a discrepancy in the knowledge and practice of informed consent by surgeons. The responses are quite different from the expected practice where informed consent is a process of dialogue between the patient and the provider [26-28]. Hence the need to address these differences with a focus on streamlining the informed consent process in the country.

In response to how informed consent is obtained, 34% of the respondents described it as follows; consent is got by the admitting nurse/doctor; by signing the consent form; obtained verbally; and implied consent. This highlighted the fact that knowledge and practice of informed consent were not well appreciated. A number of other respondents indicated that informed consent is usually obtained by the anesthesiologists or anesthetic clinical officers.

Additionally, many of the respondents interviewed said that nurses are used to obtain consent for surgery from patients. The respondents reasoned that sometimes the patients are so many and the nurses would help in consenting while the doctors did the operating which is a form of task shifting. All the above responses indicate the lack of appreciation of what constitutes adequate informed consent.

It should be stressed that informed consent should be obtained by the surgeon who is going to operate and it is a continuous process that starts as soon as the doctor meets the patient and should continue after the operation to facilitate the patient’s understanding of the procedure, benefits, anticipated risks of the operation and the post operative follow up period.

Despite the fact that there are many deficits in the informed consent process highlighted by this study, challenges associated with obtaining consent during clinical care are not limited to our setting but affect other parts of the world though the reasons could be different [22].

The magnitude of this problem thus calls for a more comprehensive approach to obtaining informed consent by development of an informed consent template that has adequate information and room for modification to facilitate the informed consent process. Additionary, refresher training and continuing education with focus on medical ethics can be made mandatory for all medical practitioners.

### Study limitations

Self reporting bias may affect the outcome of the study since some respondents may not report what they actually practice.

The high level of non-response may give a different interpretation of the findings

The alternatives in the questionnaires were not mutually exclusive which could have caused confusion to the respondents.

### Conclusions

Informed consent administration and documentation for surgical care is still inadequate at University teaching hospitals in Uganda. Surgeons need to be educated into what constitutes informed consent and the importance of adhering to such requirements.

There is need for development of an informed consent template with adequate information and room for modification to facilitate the informed consent process.

## Competing interests

The authors declare that they have no competing interest.

## Authors’ contributions

JO performed Literature search, study design, data collection, data analysis, data interpretation, drafting, writing, proof reading and approval of manuscript; CI performed Literature search study design, proof reading and approval of manuscript; WB study design, data collection, proof reading and approval; HK study design, data collection, proof reading and approval; DK study design, participated in data collection, proof reading and approval; PB proof reading and approval; IM data analysis, proof reading and approval; GN study design, data collection, proof reading and approval; EM study design, data collection, data analysis proof reading and approval. All authors read and approved content of the final manuscript.

## Authors’ information

JO is a Medical Doctor and Bioethicist, he is a senior lecturer and chair, Department of Anatomy at School of Biomedical Sciences, Makerere University; CI is an associate professor and Dean School of Biomedical Sciences, Makerere University; WB is a dental Surgeon and senior lecturer in the Department of Anatomy at Makerere; DK is a general surgeon and chair, Department of Surgery Gulu University; PB is a senior lecturer in social administration, Makarere University; HK is a dental surgeon and assistant lecturer in the Department of Anatmy, School of Biomedical Sciences Makerere University; IM is a medical doctor and an assistant lecturer in the Department of Anatomy; GN is a general surgeon and a professor of Anatomy, St. Augustine International University; EM is an orthopaedic surgeon and senior lecturer in the Department of Anatomy, School of Biomedical Sciences Makerere University.

## Pre-publication history

The pre-publication history for this paper can be accessed here:

http://www.biomedcentral.com/1472-6939/15/40/prepub

## Supplementary Material

Additional file 1 Self administered Questionnaire for Doctors. Click here for file

Additional file 2 Interview guide for in-depth interview. Click here for file
