# Supplementary material for: Informed consent practices for surgical care at university teaching hospitals: a case in a low resource setting
Source: BMC Med Ethics. 2014 May 19;15:40. doi: 10.1186/1472-6939-15-40 (PMC4068318; doi:10.1186/1472-6939-15-40)
Supplement: Additional file 1 — Self administered Questionnaire for Doctors. [file 1472-6939-15-40-S1.doc]

**Additional file 1**

**Title of the study:**

Perceptions, appropriateness and understanding of informed consent process for surgical health care in Uganda

**Self administered Questionnaire for Doctors**

1. Study number .........................................................................................................
2. Your Age: .................................................................................................................
3. Sex: ..........................................................................................................................
4. Level of education: ................................................................................................
5. Your specialization: .................................................................................................
6. Your Experience in number of years in this field of surgery: ...................................
7. Name of your Hospital: .............................................................................................
8. Average number of patients operated by you in a week: .......................................
9. Briefly explain what you understand by informed consent: ............................................................................................................................................................................................................................................................................................................................................................................................................................................................................................................................................
10. Describe how consent is obtained from your patients: ............................................................................................................................................................................................................................................................................................................................................................................................................................................................................................................................................
11. Who should obtain consent from patients for surgery? ................................
12. How much information should be provided to the patients ..................................................................................................................................................................................................................
13. Do you obtain informed consent from patients all the times: ................................................................................................................................
14. Did you obtain consent for the last surgery you performed .........................................
15. Is informed consent necessary ...................................................................................................................................................................................................................................................................
16. When should informed consent be sought ................................................................................................................................................................................................................................................................................
17. When may informed consent not be sought ............................................................
18. Do patients have a right to participate in treatment decision making: .........................................................................................................................................why?............................................................................................................................
19. Do patients have a right to refuse medical treatment: ............................................. Why?.........................................................................................................................................................................................................................
20. Doctors have a right to prescribe any treatment: ....................................................
21. Any other issues about informed consent: ........................................................................................................................................................................................................................................................................................................................................................................................................................
