# Supplementary material for: Informed consent practices for surgical care at university teaching hospitals: a case in a low resource setting
Source: BMC Med Ethics. 2014 May 19;15:40. doi: 10.1186/1472-6939-15-40 (PMC4068318; doi:10.1186/1472-6939-15-40)
Supplement: Additional file 2 — Interview guide for in-depth interview. [file 1472-6939-15-40-S2.doc]

**Additional file 2**

**Title of the study:**

Perceptions, appropriateness and understanding of informed consent process for surgical health care in Uganda

**Interview guide for in-depth interview:**

1. Briefly describe what you understand by informed consent
2. Have you ever been involved in the an informed consent process?

Probe; When?

Describe what happened

1. Who should be involved in the informed consent process?

Probe; Is it for doctors or patients

what type of patients need consent?

1. What should the informed consent process involve?

Probe; who obtains consent

For how long

Under what conditions?

1. How would you like the informed consent process to be conducted for surgical patients?

Probe; before surgery

During surgery

After surgery

All times patient meets doctor?

1. Are you satisfied with the way informed consent process is conducted in this hospital?

Probe; what is done right

What is not done right?

What should change?
